# Green tea extract for prevention of prostate cancer progression in patients on active surveillance

**DOI:** 10.18632/oncotarget.26519

**Published:** 2018-12-28

**Authors:** Nagi B. Kumar, Shohreh I. Dickinson, Michael J. Schell, Brandon J. Manley, Michael A. Poch, Julio Pow-Sang

**Affiliations:** ^1^ H. Lee Moffitt Cancer Center & Research Institute, Inc., Cancer Epidemiology, MRC/CANCONT, Tampa, FL 33612-9497, USA; ^2^ H. Lee Moffitt Cancer Center & Research Institute, Inc., Pathology Anatomic MMG, WCB-GU PROG, Tampa, FL 33612-9497, USA; ^3^ H. Lee Moffitt Cancer Center & Research Institute, Inc., Biostatics and Bioinformatics, MRC-BIOSTAT, Tampa, FL 33612-9497, USA; ^4^ H. Lee Moffitt Cancer Center & Research Institute, GU Oncology MMG, Tampa, FL 33612-9497, USA

**Keywords:** chemoprevention, prostate cancer, active surveillance, green tea catechins, prostate cancer progression

## Abstract

**Background:**

Active surveillance (AS) has evolved as a management strategy for men with low grade prostate cancer (PCa). However, these patients report anxiety, doubts about the possible progression of the disease as well as higher decisional conflict regarding selection of active surveillance, and have been reported to ultimately opt for treatment without any major change in tumor characteristics. Currently, there is a paucity of research that systematically examines alternate strategies for this target population.

**Methods:**

We conducted a review the evidence from epidemiological, *in vitro*, preclinical and early phase trials that have evaluated green tea catechins (GTC) for secondary chemoprevention of prostate cancer, focused on men opting for active surveillanceof low grade PCa.

**Results:**

Results of our review of the *in vitro*, preclinical and phase I-II trials, demonstrates that green tea catechins (GTC) can modulate several relevant intermediate biological intermediate endpoint biomarkers implicated in prostate carcinogenesis as well as clinical progression of PCa, without major side effects.

**Discussion:**

Although clinical trials using GTC have been evaluated in early phase trials in men diagnosed with High-Grade Prostatic Intraepithelial Neoplasia, Atypical Small Acinar Proliferation and in men with localized disease before prostatectomy, the effect of GTC on biological and clinical biomarkers implicated in prostate cancer progression have not been evaluated in this patient population.

**Conclusion:**

Results of these studies promise to provide a strategy for secondary chemoprevention, reduce morbidities due to overtreatment and improve quality of life in men diagnosed with low-grade PCa.

## INTRODUCTION

Prostate cancer (PCa) remains, the most common non-cutaneous malignancy among men in the United States [1]. It is estimated that there will be 164,690 new cases of PCa in the United States (US) in 2018. The American Cancer Society estimates that in 2018, 29,430 men will die from this disease [1]. With an increase in utilizing serum concentrations of prostate specific antigen (PSA) for screening and early detection of PCa over the past two decades, a significant increase in the detection of low-grade prostate cancers has been observed. Recent data has demonstrated that these low grade tumors (Gleason ≤ 6) pose little or no risk of either metastatic spread or death [2–5]. On the [2–5] other hand, over treatment is a well-recognized consequence of early detection of PCa, particularly for those men with low grade PCa who are at low risk for aggressive or lethal disease and who may be now exposed to morbidities of over treatment with little or no benefit of cancer-specific survival [4, 6]. Active surveillance (AS) has thus evolved as a recommended management strategy for men with low risk disease, providing the benefit of an individualized approach of carefully monitoring disease progression using PSA kinetics, periodic biopsies and possibly surveillance MRI, sufficient to permit timely therapeutic intervention. Using this approach, in a large cohort of men on active surveillance followed over 15 years, Klotz et al., (2015) [7] demonstrated a 98% disease-specific survival for Gleason 3+3 tumors. Other large cohort prospective studies have confirmed the safety and relative effectiveness of this approach [8].

However, several major challenges have been identified in this patient population on AS. Based on the results of the US National Cancer data base (2010-2011) [9], challenges to active surveillance include concerns about undergrading and a significant variation in criteria used to define men at low risk and eligible for active surveillance. For example, of men with prostate cancer, 39.8% vs. 28.5% vs. 10.7% were determined to be low risk and eligible for active surveillance by Klotz (least stringent) [10], D'Amico (intermediate stringent) [11] and modified Epstein criteria (Most stringent). Additionally, the percentage of men receiving active surveillance was much lower in the national sample evaluated by Maurice et al. (2015) [9], across all criteria: 6.5%, 7.4% and 12.1%, demonstrating continued overtreatment that has not substantially changed based from evidence used in the 2012 recommendations [12]. Other challenges include patient-related factors such as anxiety, depression, doubts about the possible progression of the disease as well as higher decisional conflict regarding selection of active surveillance [13, 14], reported by this cohort. Men on active surveillance have thus been reported to ultimately opt for treatment without any major change in tumor characteristics. On the other hand, men on active surveillance are a subgroup, who are highly motivated and eager to make positive lifestyle changes, including strategies to further reduce their risk of PCa progression [14–16] providing an opportunity for preventing progression through pharmacologic means [17–19]. Taking into consideration these caveats, men on active surveillance are an ideal target for chemoprevention interventions with promising agents, to potentially further reduce progression to later stage disease as well as anxiety during the period of AS. Currently, there is a paucity of research that systematically examines agents for chemoprevention men on AS, underscoring the need to identify other novel agents for prostate cancer chemoprevention in this target population that is closely monitored during the active surveillance period.

The goal for prostate cancer chemoprevention is to utilize a systematic, broad spectrum approach [20] that involves using a botanical that has been shown to have an: (a) bioavailable; (b) excellent safety profile; (c) produces robust targeting of relevant and multiple molecular pathways; and (d) modulates intermediate endpoint biomarkers implicated in clinical progress of PCa - an approach that may be more effective than agents evaluated to date.

## RESULTS

Results from epidemiological data have shown that the mortality rates of PCa is the lowest in Asia, where 20% of the world's green tea is consumed [4]. However, once these habits are abandoned upon migration to the West, including to the United States, the risk of PCa appears to increase [21]. However, case-control and cohort studies addressing the relationship between GTC consumption and PCa risk have been mixed potentially attributed to varying formulations of catechins evaluated [22, 23].

(GTCs) include (−)-epigallocatechin-3-gallate (EGCG), (−)-epicatechin (EC), (−)-epigallocatechin (EGC), and (−)-epicatechin-3-gallate (ECG). Among these compounds, laboratory studies have identified EGCG as the most potent modulator of molecular pathways thought to be relevant to prostate carcinogenesis [21–25]. EGCG has been found to affect several cancer-related proteins including Cyclin-dependent kinase inhibitor p27 (p27), Bcl-2 or Bcr-Abl oncoproteins, Bax, matrix metalloproteinases (MMP-2 and MMP-9) [26] the androgen receptor, EGF receptor, Activator proteins 1(AP1), and some cell cycle regulators. Based on these studies of GTCP in cell culture systems, Adhami et al. [26], were able to demonstrate that EGCG in GTP induces apoptosis, cell growth inhibition and cyclin kinase inhibitor Cyclin-dependent kinase inhibitor p21 (WAF)-1/p21-mediated cell cycle-dysregulation. Using cDNA microarrays, they also observed the EGCG treatment of LNCaP cells results in induction of genes that exhibit the growth-inhibitory effects and repression of genes that belong to the G-protein signaling network [26]. It is well established that the ubiquitin/proteasome pathway plays a critical function in the regulation of apoptosis. We and others have demonstrated that although there are several mechanisms by which EGCG may modulate prostate carcinogenesis, the catechin-EGCG potently and selectively inhibits the proteasome activity in intact human cells and ultimately results in the accumulation of B-α and p27 proteins, and growth arrest [27–31].

This inhibition of proteasome activity by EGCG occurred at or near physiological concentrations similar to that found in the body fluids of green tea drinkers. We have observed that Polyphenon E (a mixture of tea catechins), similar in composition to the GTC proposed in this trial, specifically inhibits the proteasomal chymotrypsin-like activity with an IC50 value of 7 μM [18]. The IC50 value for trypsin-like activity was above 100 μM, demonstrating that Polyphenon E preferentially inhibits the proteasomal chymotrypsin-like activities. Data from our studies implies that indeed, the proteasome is potentially a PCa-related molecular target of GTC. Thus the inhibition of the proteasome activity by the catechin EGCG present in GTC leading to apoptosis, ultimately impacting the prostate carcinogenesis.

We and others have reported evidence from cell culture and preclinical models that suggest that GTC inhibit proliferation and cell cycle events and induces apoptosis through multiple mechanisms including antioxidant and anti-inflammatory activity [24, 32–34]. Preclinical studies of GTCs [26, 35–39], including from our preliminary studies, have shown significant reductions in tumor size and multiplicity in the prostate cancer Trangenic adenocarcinoma of the mouse prostate (TRAMP) models. Gupta et al. [35], showed that an oral infusion of GTP extract at a human achievable dose (equivalent to six cups of green tea per day) in a TRAMP mouse model delayed primary PCa incidence, including tumor burden that was serially assessed through the study using Magnetic Resonance Imaging (MRI). In addition to observing a decrease in prostate weight by 64% of baseline, intervention with GTP inhibited serum insulin-like growth factor-I (IGF-I) and restored levels of insulin-like growth factor binding protein-3 levels (IGFBP-3). Additionally, a significant reduction in the expression of Proliferating Cell Nuclear Antigen (PCNA) proteins was observed in the prostate of GTP-fed animals compared to water-fed TRAMP mice was observed, GTP fed mice also demonstrated significant apoptosis, potentially leading to reduction in dissemination of cancer cells, ultimately inhibiting development, progression and metastasis of PCa to distant organ sites. Using the TRAMP mouse model, Suttie et al., have reported that oral administration of whole GTPs (vs. the pure form of the catechin-EGCG) was highly bioavailable [26, 29, 35–37, 39] compared to EGCG alone [39]. We evaluated the safety and effectiveness of a standardized formulation of GTP- Polyphenon E at various doses, (200, 500, and 1,000 mg/kg/day), in reducing the progression of prostate cancer in a TRAMP mouse model. We reported that in TRAMP mice treated with all doses of Polyhenon E, the number and size of PCa tumors significantly decreased compared with untreated animals. In 32 weeks, 100% of TRAMP mice that were untreated, were observed to have PCa metastasis to distant sites (8/8). On the other hand only 13% of mice (2/16) treated with high-dose (1000 mgs/Kg/day) Polyphenon E were observed to PCa metastasis to distant sites. Our team, in addition, observed that treatment with Polyphenon E significantly inhibited metastasis in TRAMP mice in a dose-dependent manner (P = 0.0003). Thus, a 32 week treatment with standardized formulation of GTC was well tolerated with no evidence of toxicity in C57BL/6J mice [38].

Early phase trials completed in the past decade [40–51] have shown that GTC containing doses of the catechin, EGCG ranging from 200 to 1200 mg a day is well tolerated. Although most catechins as measured in plasma appeared in low to non-detectable concentrations, EGCG appears to be the most bioavailable in the plasma [52–54], with greater bioavailability observed in a fasting state [45]. However, greater gastrointestinal toxicities have been reported when GTC is consumed in a fasting state compared when consumed in a fed state. Similarly, the bioavailability and tolerance to a multiple dosing schedule compared to a single daily dose of EGCG has been reported in phase II trials [32, 40, 41, 48]. Phase I/II [40–51] studies of less than 3 month duration, have demonstrated bioavailability as well as drug effects of GTC at doses ranging from 200-1200 mg EGCG in GTC per day. Findings from our study (decaffeinated GTC 200 mgs BID of EGCG) and Bettuzzi et al. [40, 48], (600 mgs EGCG per day) have demonstrated that a daily intake of standardized formulation administered for 12 months, with food (non-fasting), accumulates in plasma, reduces serum PSA [48] and cumulative rate of progression to PCa with no toxicities. However, these were primary chemoprevention trials with subjects diagnosed with HGPIN or ASAP and not cancer. In three (3) randomized trials [32, 33, 55] using GTC as a standardized supplement or beverage with EGCG content ranging from 500- 800 mgs) for a maximum duration of intervention of 6 weeks administered prior to prostatectomy in men diagnosed with localized PCa, GTC were well tolerated with only mild toxicities observed. Most adverse events that have been reported with GTC have included mild nausea observed at doses of 1200 mgs EGCG dose/day and when patients were fasting. This has informed future clinical trials to use GTP formulations containing no more than 800 mg of EGCG per day with instructions to patients to consume the catechins with food. Others have shown that GTC administered for 4 weeks did not after drug metabolizing enzymes [44, 56] including CYP2D6 or CYP3A4 activity [57].

Virtually all active surveillance programs continue to monitor PSA serially. PSA kinetic measurements such as PSA velocity (PSAV) and PSA doubling time Prostate specific antigen doubling time (PSADT) have been extensively evaluated as predictors of progression [19, 58]. In several active surveillance programs, PSA kinetics was historically used as a trigger for intervention with a significant association between PSA kinetics and progression to treatment [59, 60]. However, studies have shown that PSA kinetics are not a reliable predictor of biopsy reclassification during active surveillance [61, 62]. As a result, PSA kinetics is now considered trigger for further diagnostic evaluation rather than as a trigger for intervention [63]. Patel et al. [64], showed that men with multiple successive PSAV measurements >0.4 ng/ml/year have a significantly greater risk of biopsy reclassification beyond the first 2 years of active surveillance [65]. Thus, men with stable disease on active surveillance for several years may benefit from the use of PSA kinetics [64, 66, 67]. Other PSA measurement such as Prostate Specific Antigen Density (PSAD) at the time of diagnostic biopsy can be used to predict progression and is predictive of biopsy reclassification at subsequent surveillance [68], and can thus be used to predict progression during active surveillance. Approved by the Food and Drug Administration (FDA) in 2012 as an aid in early PCa detection [69]. The Prostate Health Index (phi) is an adjunctive PSA-based measurement combining total, free, and [−2] proPSA using a mathematical formula and has been shown to outperform PSA and free PSA for identifying clinically significant PCa [70], and a predictor of biopsy reclassification [34, 71]. Additional studies are needed to identify how phi can be best utilized, possibly in conjunction with imaging, to help monitor men during active surveillance [61–63, 67]. Bettuzzi and colleagues reported a significant reduction in prostate cancer in men with HGPIN randomized to receive one-year of EGCG [40, 41]. We reported the results of a randomized, placebo-controlled phase II clinical trial examining the safety and effectiveness of Polyphenon E (PolyE), a mixture of GTCs, containing 400 mg (−)-epigallocatechin-3-gallate (EGCG) per day. We recruited and treated 97 men diagnosed with HGPIN and/or atypical small acinar proliferation (ASAP). We observed a greater number of men progressed to prostate cancer in 1 year in the placebo arm compared to men treated with Polyphenon E (5 of 49 (Poly E) versus 9 of 48 (placebo), P = 0.25). Among men with HGPIN without ASAP at baseline, examining a pre-specified secondary endpoint of cumulative rate of progression from HGPIN to prostate cancer or ASAP, we observed a significantly greater number of men progressing to prostate cancer in the Poly E treated arm compared to the placebo (3 of 26 (PolyE) versus 10 of 25 (placebo), P < 0.024). We also observed a a significant decrease in serum PSA with PolyE treatment [−0.87 ng/mL; 95% confidence intervals (CI), −1.66 to −0.09] compared to placebo [48, 72]. We observed a significant decrease in serum PSA in men on the PolyE arm [−0.87 ng/mL; 95% confidence intervals (CI), −1.66 to −0.09] compared to the placebo arm [48, 72]. Similar results were observed by Henning, et al. who also demonstrated statistically significant reduction in serum PSA with GTC. (P = 0.04) [32]. In addition to reduction of serum PSA, McLarty et al. [33], showed a significant reduction in Hepatocyte Growth Factor (HGF), and Vascular Endrothelial Growth Factor (VEGF) after treatment with GTC containing a doe of 800 mgs EGCG (Polyphenon E) targeting PCa patients, with no elevation of liver enzymes.

In summary, epidemiological, laboratory and early phase clinical trials over the past two decades have demonstrated that formulations of GTC containing over 50% catechins in the form of EGCG administered to men at high risk or men with localized PCa, is bioavailable in plasma, reduces serum PSA, modulates proliferative and apoptotic intermediate endpoint biomarkers implicated in prostate carcinogenesis and the cumulative rate of progression to prostate cancer with no toxicities, including liver enzymes. Standardized formulations of GTC thus appears to be promising agents for prostate cancer chemoprevention.

## DISCUSSION

The current recommendations to manage patients with low grade PCa is active surveillance while being carefully monitored for disease progression using PSA kinetics, periodic biopsies for histologic progression, sufficient to permit timely therapeutic intervention. However, concerns about under-grading, variations in criteria for active surveillance eligibility, patient-related factors such as anxiety, depression, doubts about the possible progression of the disease as well as higher decisional conflict regarding selection of active surveillance result in men on active surveillance ultimately opt for treatment without any major change in tumor characteristics. On the other hand, men on active surveillance are a subgroup, who are highly motivated and eager to make positive lifestyle changes, including strategies to further reduce their risk of PCa progression-providing an opportunity for preventing progression through pharmacologic means.

Previous chemoprevention strategies targeting men on active surveillance have included large phase III trials with 5-alpha-reductase inhibitors, finasteride and dutasteride [19, 73, 74]. Although these agents significantly reduced the risk of prostate cancer progression, their use was also associated with increased detection of high-grade disease, severely limiting their clinical adoption [74]. Another large, phase III prostate cancer prevention clinical trial showed no benefit for long-term supplementation with the trace element Se, given in the form of selenomethionine, or vitamin E, either individually or in combination. However, a significant increase in prostate cancer was observed among men randomized to receive vitamin E alone [75]. Currently, strategies using dietary interventions (The Men's Eating and Living (MEAL) Study: A Randomized Trial of Diet to Alter Disease Progression in Prostate Cancer Patients on Active Surveillance) [76], as well as, pomegranate fruit extract (https://clinicaltrials.gov/ Identifier: NCT02095145) targeting the active surveillance population are ongoing with results of these trials pending. However, to date, there is minimal evidence available of any one agent or strategy that has been found to be effective for chemoprevention for men on active surveillance for PCa. There is thus a paucity of research that systematically examines agents for chemoprevention men on active surveillance that are available, underscoring the need to identify and test other novel agents for prostate cancer chemoprevention in this target population. Although green tea catechins have been examined in clinical trials targeting men with HGPIN, ASAP and in men prior to prostatectomy, to-date, the effect of GTP on biological and clinical intermediate endpoint biomarkers implicated in prostate cancer progression have not been evaluated in men on active surveillance. Based on the *in vitro*, preclinical and phase I-II trials, green tea catechins, GTC appear be a promising agent for prostate cancer chemoprevention, establishing the evidence needed to be further test this agent in the active surveillance population, for whom, currently, there are no options for reducing their risk.

## MATERIALS AND METHODS

The goal of the current review is to evaluate the current evidence from epidemiological, *in vitro*, preclinical and early phase trials completed by our team and others that have established the evidence needed for further development of Green Tea C (GTC) for secondary chemoprevention of prostate cancer targeting men on active surveillance for prostate cancer. We searched eligible studies conducted in the past 2 decades up to October 2018 in the PUBMED, MEDLINE and EMBASE. We included *in vitro*, preclinical and all prospective, controlled interventional studies and observational studies, which evaluated the associations between green tea catechin consumption and risk of prostate cancer incidence, modulation of intermediate endpoint biomarkers implicated in prostate carcinogenesis, prostate cancer progression or that reported on cancer mortality. The review was limited to a GTC in prostate carcinogenesis and not other additional agents or diseases, including other cancers. A minimum of two investigators independently reviewed the data according to the established inclusion criteria. Additionally, the methodological quality of the studies was assessed by all authors specifically as it related to modulation of prostate carcinogensis by green tea catechins.

## CONCLUSIONS

Systematically evaluating the effectiveness and safety of novel agents such as green tea catechins, which have been well characterized in laboratory and early phase clinical trials, may inform the development of phase III clinical trials and ultimately provide strategies for chemoprevention in men on active surveillance for prostate cancer, for whom, currently, there are no options for reducing their risk.
